# Treatment of acute ankle ligament injuries: a systematic review

**DOI:** 10.1007/s00402-013-1742-5

**Published:** 2013-05-28

**Authors:** Wolf Petersen, Ingo Volker Rembitzki, Andreas Gösele Koppenburg, Andre Ellermann, Christian Liebau, Gerd Peter Brüggemann, Raymond Best

**Affiliations:** 1 Department of Orthopaedic and Trauma Surgery, Martin Luther Hospital, Caspar Theyß Strasse 27-31, Grunewald, 14193 Berlin, Germany; 2 Asklepios Harzkliniken GmbH, Fritz-König-Stift, Bad Harzburg, Germany; 3 Cross Klinik Basel, Olympic Medical Center, Basel, Switzerland; 4 Arcus Sportklinik, Pforzheim, Germany; 5 Deutsche Sporthochschule Köln, Institut für Biomechanik, Cologne, Germany; 6 Sportorthopädische Klinik Tübingen, Tübingen, Germany

**Keywords:** Chronic ankle instability, Ankle brace, External support, Surgical treatment, Balance training

## Abstract

**Background:**

Lateral ankle sprains are common musculoskeletal injuries.

**Objectives:**

The objective of this study was to perform a systematic literature review of the last 10 years regarding evidence for the treatment and prevention of lateral ankle sprains.

**Data source:**

Pubmed central, Google scholar.

**Study eligibility criteria:**

Meta-analysis, prospective randomized trials, English language articles.

**Interventions:**

Surgical and non-surgical treatment, immobilization versus functional treatment, different external supports, balance training for rehabilitation, balance training for prevention, braces for prevention.

**Methods:**

A systematic search for articles about the treatment of lateral ankle sprains that were published between January 2002 and December 2012.

**Results:**

Three meta-analysis and 19 articles reporting 16 prospective randomized trials could be identified. The main advantage of surgical ankle ligament repair is that objective instability and recurrence rate is less common when compared with non-operative treatment. Balancing the advantages and disadvantages of surgical and non-surgical treatment, we conclude that the majority of grades I, II and III lateral ankle ligament ruptures can be managed without surgery. For non-surgical treatment, long-term immobilization should be avoided. For grade III injuries, however, a short period of immobilization (max. 10 days) in a below knee cast was shown to be advantageous. After this phase, the ankle is most effectively protected against inversion by a semi-rigid ankle brace. Even grades I and II injuries are most effectively treated with a semi-rigid ankle brace. There is evidence that treatment of acute ankle sprains should be supported by a neuromuscular training. Balance training is also effective for the prevention of ankle sprains in athletes with the previous sprains. There is good evidence from high level randomized trials in the literature that the use of a brace is effective for the prevention of ankle sprains.

**Conclusion:**

Balancing the advantages and disadvantages of surgical and non-surgical treatment, we conclude that the majority of grades I, II and III lateral ankle ligament ruptures can be managed without surgery. The indication for surgical repair should be always made on an individual basis. This systematic review supports a phase adapted non-surgical treatment of acute ankle sprains with a short-term immobilization for grade III injuries followed by a semi-rigid brace. More prospective randomized studies with a longer follow-up are needed to find out what type of non-surgical treatment has the lowest re-sprain rate.

## Introduction

Ankle sprains are one of the most common musculoskeletal injuries. In all sports injuries, the rate of ankle sprains ranges from 15 to 20 % [12, 23].

The most common injury mechanism is a combination of inversion and adduction of the foot in plantar flexion (supination). This injury mechanism can cause damage to the lateral ankle ligaments [3]. Injury of the anterior talofibular ligament with intact medial ligaments leads to anterolateral rotary instability [8]. Additional transection of the calcaneofibular ligament adds a tilting of the talus (talar tilt) [8].

Ankle ligament sprains are usually graded on the basis of severity [3]. Grade I is a mild stretching of the ligaments without macroscopic rupture or joint instability. Grade II (moderate) is a partial rupture of the ligament with moderate pain and swelling. There are functional limitations and a slight to moderate instability. Typically, patients present with problems in weight bearing [4]. Grade III (severe) is a complete ligament rupture with marked pain, swelling, hematoma and pain. In grade III injuries, there is a marked impairment of function with instability.

Biological ligament healing can be divided into three different phases [21]: (1) inflammatory phase (until 10 days after trauma), (2) the proliferation phase (4th–8th week) and (3) the remodelling or maturation phase (until 1 year after trauma). The duration of the different phases may individually vary.

Many treatment options have been suggested: surgery, immobilization, functional treatment with bandages, tape or different braces, balance training. Today, most authors recommend non-surgical treatment for lateral ankle sprains.

Nevertheless, many studies have shown that ankle sprains are more serious than commonly believed since many patients develop chronic problems after injury [17, 55, 57]. The symptoms, include chronic pain, recurrent swelling, and chronic instability [17, 55]. In addition, there is strong evidence that within 1 year after injury, athletes have twice the risk of a recurrent ankle sprain [1, 12, 34]. Interestingly, Malliaropoulos [29] found that low-grade acute lateral ankle sprains result in a higher risk of reinjury than high-grade acute lateral ankle sprains.

The high rate of failure after ankle sprain treatment might be explained by overlooked associated lesions, such as syndesmosis or cartilage injuries [17]. Another cause may be inappropriate treatment with regard of the different injury grades and healing phases.

To find out which treatment option is the most appropriate one, we have performed a systematic review of the literature published the last 10 years. This review should answer the following research questions:

1. Is there evidence for surgical or non-surgical treatment of acute ankle sprains?
2. Is there evidence for functional treatment or immobilization?
3. What is the most effective type of external stabilization for the treatment of acute ankle sprain?
4. Is there any evidence for neuromuscular training for rehabilitation of acute ankle sprains?
5. Is there any evidence for neuromuscular training for the prevention of ankle sprains?
6. Is there any role for prophylactic bracing?

## Methods

We conducted a comprehensive literature search using the MEDLINE database and Google scholar to identify peer reviewed articles about the treatment of lateral ankle sprains according to the PRISMA statement [37].

For the systematic review, different combinations of keywords were utilized: (1) ankle sprain, (2) ankle ligament injury, (3) ankle sprain and rehabilitation, (4) ankle sprain and surgical treatment, (5) ankle sprain and functional treatment, (6) ankle sprain and external support, (7) ankle sprain and neuromuscular training.

After each article identified in Pubmed, the “see all” button for related article was activated. Furthermore, the reference lists of the identified articles were screened for relevant publications.

Only contemporary articles published within the last 10 years (first January 2002 to December 2012) were considered for review. The reason for this approach is that many earlier than 2002 published studies were already included in the meta-analyzes. The search was restricted to English language articles. We excluded articles which considered management of ankle fractures, syndesmosis lesions or dislocations. If a prospective randomized trial was already included in a meta-analysis, this trial was also excluded.

The patient selection was limited to adults equal to or greater than 16 years of age. We only considered articles of level I evidence according to the Agency for Healthcare Research and Quality [47]: meta-analysis and randomized controlled trial (RCT). Data from studies of lower evidence levels were only considered when these were included in meta-analysis. Cohort studies, case series, retrospective studies, case reports, expert opinion and anecdotal evidence were not considered.

If a study of interest was found the abstract was studied to find out if any of the exclusion criteria applied. If the study was eligible the full text article was studied. The article should be suited to answer one of the six research questions stated at the end of the introduction.

## Results

**Fig. 1:**
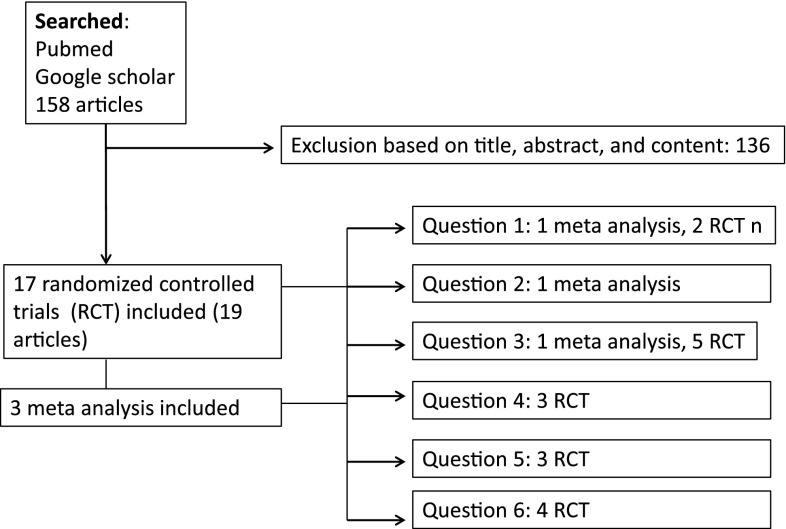
Flow chart for selecting articles to be included in the systematic review to answer our three questions. Article reference numbers are superscripted

One hundred fifty-eight articles could be identified and 136 articles had to be excluded (Fig. 1). Three meta-analyzes and 17 RCTs were included in the analysis.

### Meta-analysis about treatment options for acute ankle sprains published between 2002 and 2012

We identified three different meta-analysis about the treatment of ankle sprains. These three meta-analysis are summarized in Table 1.

**Table 1:** Randomized trials about non-operative treatment with external support between 2002 and 2012

References	Number of trials and patients	Treatment options	Results	Authors conclusions
Kerkhoffs et al. [26]	20 trials were included. These involved a total of 2,562 mostly young active adult males.	Surgical vs. non-surgical	“The findings of statistically significant differences in favour of the surgical treatment group for the four primary outcomes (non-return to pre-injury level of sports; ankle sprain recurrence; long-term pain; subjective or functional instability) when using the fixed-effect model were not robust when using the random-effects model, nor on the removal of one low quality (quasi-randomized) trial that had more extreme results. The functional implications of the statistically significantly higher incidence of objective instability in conservatively treated trial participants are uncertain. There was some limited evidence for longer recovery times, and higher incidences of ankle stiffness, impaired ankle mobility and complications in the surgical treatment group”	“There is insufficient evidence available from randomized controlled trials to determine the relative effectiveness of surgical and conservative treatment for acute injuries of the lateral ligament complex of the ankle. High quality randomized controlled trials of primary surgical repair versus the best available conservative treatment for well-defined injuries are required”
Kerkhoffs et al. [24]	Twenty-one trials involving 2,184 participants	Functional treatment vs. immobilisation	“Statistically significant differences in favour of functional treatment when compared with immobilization were found for seven outcome measures: Return to sports rate, time to return to sports, return to work rate, time to return to work, swelling, and satisfaction with treatment (RR 1.83, 95 % CI 1.09 to 3.07). No significant differences between varying types of immobilization, immobilization and physiotherapy or no treatment were found, apart from one trial where patients returned to work sooner after treatment with a soft cast. In all analyses performed, no results were significantly in favour of immobilization”	“Functional treatment appears to be the favourable strategy for treating acute ankle sprains when compared with immobilization. However, these results should be interpreted with caution, as most of the differences are not significant after exclusion of the low quality trials. Many trials were poorly reported and there was variety amongst the functional treatments evaluated”
Kerkhoffs et al. [25]	Nine trials involving 892 participants were included	Elastic bandage, tape, semi rigid support	“Lace-up ankle support had significantly better results for persistent swelling at short-term follow up when compared with semi-rigid ankle support; elastic bandage; and to tape. Use of a semi-rigid ankle support resulted in a significantly shorter time to return to work when compared with an elastic bandage; one trial found the use of a semi-rigid ankle support saw a significantly quicker return to sport compared with elastic bandage and another trial found fewer patients reported instability at short-term follow-up when treated with a semi-rigid support than with an elastic bandage. Tape treatment resulted in significantly more complications, the majority being skin irritations, when compared with treatment with an elastic bandage”	“The use of an elastic bandage has fewer complications than taping but appears to be associated with a slower return to work and sport, and more reported instability than a semi-rigid ankle support. Lace-up ankle support appears to be effective in reducing swelling in the short-term compared with semi-rigid ankle support, elastic bandage and tape. However, definitive conclusions are hampered by the variety of treatments used, and the inconsistency of reported follow-up times. The most effective treatment, both clinically and in costs, is unclear from currently available randomized trials”

Kerkhoff et al. [26] analyzed trials comparing surgical and non-operative treatment. This meta-analysis showed statistically significant differences in favour of the surgical treatment for return to pre-injury level of sports; ankle sprain recurrence; long-term pain; subjective or functional instability when using the fixed-effect model. These differences were not robust when using the random-effects model, nor on the removal of one low quality (quasi-randomized) trial that had more extreme results.

The functional implications of the statistically significantly higher incidence of objective instability in conservatively treated trial participants are uncertain. There was some limited evidence for longer recovery times, and higher incidences of ankle stiffness, impaired ankle mobility and complications in surgically treated patients.

In another meta-analysis, Kerkhoffs et al. [24] compared studies about functional treatment and immobilization. This study showed statistically significant differences in favour of functional treatment when compared with immobilization for seven outcome parameters: return to sports rate, time to return to sports, return to work rate, time to return to work, swelling, and satisfaction with treatment.

In a third meta-analysis, Kerkhoffs et al. [25] compared the effect of different types of external support for non-operative treatment of ankle sprains. This study showed that lace-up ankle support had significantly better results for persistent swelling at short-term follow-up when compared with semi-rigid ankle support; elastic bandage; and to tape. The use of a semi-rigid ankle support resulted in a significantly lower rate of instability, shorter time to return to work and return to sports when compared with an elastic bandage. Tape treatment resulted in significantly more complications, the majority being skin irritations, when compared with treatment with an elastic bandage.

### Randomized controlled trials about surgical versus non-surgical treatment

We found two randomized controlled trials analyzing the effect of surgical versus non-surgical treatment for acute ankle sprain. These trials are summarized in Table 2.

**Table 2:** Randomized controlled trials about surgical versus non-surgical treatment for treatment of acute ankle sprains published between 2002 and 2012

References	Follow up	Treatment groups	Results	Authors conclusions
Takao et al. [51]	2 years	Functional treatment alone Surgical repair followed by functional treatment	Mean JSSF scores were 95.6 points in the functional treatment group and 97.5 points in the surgical group. Talar tilt angles (stress radiography): Functional treatment -1.1° ± 1.5° and 3.6 ± 1.6 mm, and 0.8° ± 0.9° and 3.2 ± 0.8 mm in the surgical group In the functional group, 8 cases showed fair to poor results, with JSSF scores below 80 points and instability at 2 years after injury. In the surgical group, 9 cases (9.4 %) showed dorsum foot pain along the superficial peroneal nerve, which disappeared within a month. Time to return to full athletic activity without any external supports; Functional group: 16.0 weeks, surgical group: 10.1 weeks in group RF (*P* < 0.0001)	“Non-operative functional treatment alone and functional treatment after primary surgical repair showed similar overall results after acute lateral ankle sprain, but functional treatment alone had an approximately 10 % failure rate and a slower return to full athletic activity. The authors recommend that treatment be tailored to suit each individual athlete”
Pihlajamäki et al. [44]		Surgical treatment followed by 6 weeks cast treatment Functional treatment.	No difference in preinjury activity level, ankle scores and stress X rays. Prevalence of reinjury: 1/15 in the surgical group, 7/18 in the functional treatment group (risk difference: 32 %) Grade-II osteoarthritis (observed on MRI) of 4 of the 15 surgically treated patients and in none of the eighteen functionally treated patients (risk difference: 27 %)	“These findings indicate that, in terms of recovery of the preinjury activity level, the long-term results of surgical treatment of acute lateral ligament rupture of the ankle correspond with those of functional treatment. Although surgery appeared to decrease the prevalence of reinjury of the lateral ligaments, there may be an increased risk for the subsequent development of osteoarthritis”

Pihlajamäki et al. [44] examined suture repair followed by 6 weeks cast treatment vs. functional treatment in patients with a grade III injury. Functional treatment consisted of the use of an Aircast ankle brace for 3 weeks. In this study, the prevalence of reinjury was 1 of 15 in the surgical group and 7 of 18 in the functional treatment group. There was no difference in the ankle score and in anterior drawer and talar tilt as measured by stress radiography. The rate of grade II osteoarthritis detected on MRI was higher in surgically treated patients when compared with functional treatment.

Takao et al. [51] examined patients after suture repair of the lateral ankle ligaments followed by functional treatment and functional treatment alone. In this study, there was also no difference could be detected in the mean results of the clinical score and ankle stability examined with stress radiography. However, in the group with functional treatment alone 8 of 132 patients suffered from instability after 2 years follow-up in contrast to none in the surgical group. Patients after surgical repair returned significantly quicker to full athletic than patients after functional treatment alone.

### Randomized controlled trials about external support

We found 6 articles reporting 5 randomized controlled trials analyzing the effect of different types of external support for the treatment of acute ankle sprain. These studies are summarized in Table 3. Two of these studies reported results from one single trial (CAST trial).

**Table 3:** Randomized trials about non-operative treatment with external support between 2002 and 2012

References	Follow up	Treatment groups	Results	Conclusions
Boyce et al. [7]	48–72 h, 10 days, and 1 month	Elastic support bandage (N: 25),Aircast ankle brace (N: 25)	No significant differences in pain scores (mean 6.2 and 5.8, respectively) Karlsson score was significantly higher in the Aircast ankle cast group than in the elastic bandage group at 10 days and 1 month No difference between the groups in the secondary outcome measures (swelling, *P* = 0.09; pain, *P* = 0.07)	“The use of an Aircast ankle brace for the treatment of lateral ligament ankle sprains produces a significant improvement in ankle joint function at both 10 days and 1 month compared with standard management with an elastic support bandage”
Cooke et al. (CAST trial) [9]	4 weeks, 3 and 9 months	Tubular bandage, 10 days below knee cast, Aircast ankle brace or Bledsoe boot	The below knee cast offered a small but statistically significant benefit at 4 weeks in terms of pain, foot- and ankle-related quality of life and the physical component of the SF-12 At 12 weeks the below knee cast was significantly better than tubular bandage in terms of pain, activities of daily living, sports and QoL, and the Aircast brace was better only in terms of ankle-related QoL and mental health The Bledsoe boot conferred no significant advantage over tubular bandage By 9 months there were no significant differences. Cost-utility analysis: Aircast brace and below knee cast (were more cost-effective than the Bledsoe boot	“The below knee cast and the Aircast brace offered cost-effective alternatives to tubular bandage for acute severe ankle sprain, the former having the advantage in terms of overall recovery at 3 months. As there were no differences in long-term outcome, practitioners should consider likely compliance and acceptability to patients when choosing a brace”
Lamb et al. (CAST trial) [27]	4 weeks, 3 and 9 months	Tubular bandage, 10 days below knee cast, Aircast ankle brace or Bledsoe boot	More rapid recovery with below-knee cast had than with tubular compression bandage Benefits in quality of ankle function, pain, symptoms, and activity with the cast compared with tubular compression bandage at 3 months Difference in quality of ankle function between Aircast brace and tubular compression bandage There were no significant differences between tubular compression bandage and the other treatments at 9 months	“A short period of immobilization in a below-knee cast or Aircast results in faster recovery than if the patient is only given tubular compression bandage. We recommend below-knee casts because they show the widest range of benefit”
Lardenoye et al. (2012) [28]		100 patients with grade II and III sprains: (1) tape and (2) semi-rigid ankle brace, both for 4 weeks. Post-injury physical and proprioceptive training in both groups	Patient-reported comfort and satisfaction during treatment with a semi-rigid brace was significantly increased. The rate of skin complication in this group was significantly lower compared to the tape group (14.6 % vs. 59.1 %, *P* < 0.0001). Functional outcome of the ankle joint was similar between the two treatment groups, as well as reported pain	“Treatment of acute ankle sprain with semi-rigid brace leads to significantly higher patient comfort and satisfaction, both with similar good outcome”
Beynnon et al. [5]		AIR-Stirrup brace alone, AIR-Stirrup brace with elastic wrap, 10 days below knee cast followed by bracing	Grade I sprains: Air-Stirrup brace combined with an elastic wrap returned subjects to normal walking and stair climbing in half the time required for those treated with the Air-Stirrup brace alone form those treated with an elastic wrap alone. Grade II sprains: The Air-Stirrup brace combined with the elastic wrap allowed patients to return to normal walking and stair climbing in the shortest time interval. Grade III sprains: The Air-Stirrup brace or a walking cast for 10 days followed by bracing returned subjects to normal walking and stair climbing in the same time intervals. The 6-month follow-up of each sprain severity group revealed no difference between the treatments for frequency of reinjury, ankle motion, and function	“Treatment of first-time grade I and II ankle ligament sprains with the Air-Stirrup brace combined with an elastic wrap provides earlier return to preinjury function compared to use of the Air-Stirrup brace alone, an elastic wrap alone, or a walking cast for 10 days”
Sultan et al. [49]		Tubigrip below knee elastic stockings	By 8 weeks, the mean AOFAS and SF12v2 scores were significantly improved by ES at 99 (8.1) and 119 (118–121) compared with 88 [11] and 102 (99–107) with Tubigrip (*P* < 0.001)	“Elastic compression improves recovery following ankle sprain”

Boyce et al. [7] showed that Karlsson score was significantly higher in the Aircast ankle brace group than in the elastic bandage group at 10 days and 1 month.

The results of the CAST trial [9, 27] showed that a short period of immobilization in a below-knee cast or treatment with a semi-rigid orthosis results in faster recovery than if the patient is only given tubular compression bandage. There was no difference between below knee cast, semi-rigid orthosis and tubular compression bandage at 9 month follow-up.

Lardenoye et al. [28] compared tape versus a semi-rigid orthosis. Functional outcome and pain was similar between the two treatment groups. Patient-reported comfort and satisfaction during treatment was significantly increased and the rate of skin complication was significantly lower in the brace group.

Bennyon et al. [5] examined patients of grades I, II, and III injuries. For grades I and II injuries, a semi-rigid ankle brace (Air-Stirrup^®^) combined with an elastic wrap returned patients quicker to normal walking and stair climbing than a semi-rigid brace alone. For grade III sprains, the Air-Stirrup brace or a walking cast for 10 days followed by bracing returned subjects to normal walking and stair climbing in the same time intervals. The 6-month follow-up of each sprain severity group revealed no difference between the treatments for frequency of reinjury, ankle motion, and function.

Sultan et al. [49] compared elastic stockings with a Tubigrip bandage. These authors found that elastic compression improves recovery following ankle sprain.

### Randomized controlled trials about the effect of training for treatment of acute ankle sprains

We found four publications about three randomized controlled trials analyzing the effect of neuromuscular training for the treatment of acute ankle sprains [6, 22, 56, 61]. These studies are summarized in Table 4. Two of these articles were reports about one trial [22, 61].

**Table 4:** Randomized trials about the effect of training for the treatment of acute ankle sprains between 2002 and 2012

References	Treatment groups	Results	Conclusions
Hupperets et al. (2009) (2Bfit Study) [22]	Both groups (N: 522) received treatment according to usual care. Athletes allocated to the intervention group additionally received an eight-week home-based proprioceptive training program	During the 1 year follow-up, 145 athletes reported a recurrent ankle sprain: 56 (22 %) in the intervention group and 89 (33 %) in the control group	“The use of a proprioceptive training program after usual care of an ankle sprain is effective for the prevention of self-reported recurrences. This proprioceptive training was specifically beneficial in athletes whose original sprain was not medically treated”
Verhagen et al. (2011) (2Bfit Study)[61]	Randomized controlled trial (RCT) involving 522 athletes who sustained a lateral ankle sprain allocated to either an intervention or control group who were followed prospectively for one year	Twenty-three percent of the RCT intervention group indicated to have fully adhered with the neuromuscular training program. A per protocol analysis only considering fully adherent athletes and control athletes, showed a Hazard Ratio of 0.18 (95 % CI: 0.07–0.43). Significantly fewer recurrent ankle sprains were found in the fully adherent group compared to the group that was not adherent (relative risk = 0.63; 95 % CI: 0.43–0.99)	“A PP analysis on fully adherent athletes versus control group athletes showed that the established intervention effect was over threefold higher compared to an earlier intention-to-treat based analysis approach. This shows that outcomes of intervention studies are heavily biased by adherence to the allocated intervention”
Bleakley et al. [6]	102 participants with grade I and II ankle sprain were randomized to an accelerated intervention with early therapeutic exercise (exercise group) or a standard protection, rest, ice, compression, and elevation intervention (standard group)	An overall treatment effect was in favour of the exercise group (*P* = 0.0077); this was significant at both week 1 and week 2. Activity level was significantly higher in the exercise group as measured by time spent walking, step count and time spent in light intensity activity. The groups did not differ at any other time point for pain at rest, pain on activity, or swelling. The reinjury rate was 4 % (two in each group)	“An accelerated exercise protocol during the 1 week after ankle sprain improved ankle function; the group receiving this intervention was more active during that week than the group receiving standard care”
Van Rijn et al. (2007) [55]	102 patients were enrolled and randomized to either conventional treatment alone or conventional treatment combined with supervised exercise	There was no significant difference between treatment groups concerning subjective recovery or occurrence of resprains after 3 months and 1 year of follow-up	“Conventional treatment combined with supervised exercises compared to conventional treatment alone during the 1 year after an acute lateral ankle sprain does not lead to differences in the occurrence of re sprains or in subjective recovery”

In the 2BFit study there was a significant reduction in resprains in the training group [22, 61]. Two studies found that conventional treatment of ankle sprains combined with supervised exercises does not lead to differences in the occurrence of resprains [6, 60]. However, one of these studies showed that patients, who received a balance board training were more active [6].

### Randomized controlled trials about the effect of training for prevention of ankle sprain

Three prospective randomized studies about the effect of balance board training for the prevention of ankle sprains in athletes could be identified [30, 58, 60]. Two studies report about the same trial (Table 5).

**Table 5:** Randomized trials about the effect of training for prevention of ankle sprains between 2002 and 2012

References	Treatment groups	Results	Conclusions
^a^Verhagen et al. [58]	Balance board (N: 419) vs. normal training (N:339) in volleyball	Significantly fewer ankle sprains in the intervention group were found compared to the control group (risk difference = 0.4/1000 playing hours; 95 % confidence interval, 0.1–0.7). A significant reduction in ankle sprain risk was found only for players with a history of ankle sprains.	“Use of proprioceptive balance board program is effective for prevention of ankle sprain recurrences”
^a^Verhagen et al. [60]	Balance board (N: 419) vs. normal training (N:339) in volleyball	The total costs per player (including the intervention material) were significantly higher in the intervention group (36.99 (93.87)) than in the control group (18.94 (147.09)). The cost of preventing one ankle sprain was approximately 444.03. Sensitivity analysis showed that a proprioceptive balance board training program aimed only at players with previous ankle sprains could be cost effective over a longer period of time	“Positive effects of the balance board program could only be achieved at certain costs. However, if broadly implemented, costs associated with the balance board program would probably be lower”
McGuine et al. [30]	765 high school soccer and basketball players (523 girls and 242 boys) balance training program vs. standard conditioning exercises	The rate of ankle sprains was significantly lower for subjects in the intervention group (6.1 %, 1.13 of 1,000 exposures vs. 9.9 %, 1.87 of 1,000 exposures; *P* = 0.04). Athletes with a history of an ankle sprain had a 2-fold increased risk of sustaining a sprain (risk ratio, 2.14), whereas athletes who performed the intervention program decreased their risk of a sprain by one half (risk ratio, 0.56). The ankle sprain rate for athletes without previous sprains was 4.3 % in the intervention group and 7.7 % in the control group, but this difference was not significant (*P* = 0.059)	“A balance training program will significantly reduce the risk of ankle sprains in high school soccer and basketball players”

^a^Same study

Both trials showed that a balance training program significantly reduces the risk of ankle sprains only in the subgroup of athletes with a previous sprain [30, 58, 60]. A subsequent economic analysis of Verhagens trial showed that balance training could be cost-effective if it is aimed only at players with the previous ankle sprains [60].

### Randomized controlled trials about the preventive effect of braces

We identified four randomized controlled trials about the preventive effect of ankle braces published between 2002 and 2012 (Table 6).

**Table 6:** Randomized trials about the role of external ankle support for the prevention of sprains

References	Treatment groups	Results	Conclusions
McGuine et al. [32]	Group treated with a lace-up ankle brace and non-braced control group in high school basketball	The rate of acute ankle injury (per 1,000 exposures) was 0.47 in the braced group and 1.41 in the control group (Cox hazard ratio [HR] 0.32; 95 % confidence interval [CI] 0.20, 0.52; *P* < 0.001)	Use of lace-up ankle braces reduced the incidence but not the severity of acute ankle injuries in male and female high school basketball athletes both with and without a previous history of an ankle injury
McGuine et al. [31]	Group treated with a lace-up ankle brace and non-braced control group in high school football	The rate of acute ankle injury (per 1000 exposures) was 0.48 in the braced group compared with 1.12 in the control group	Players who used lace-up ankle braces had a lower incidence of acute ankle injuries but no difference in the incidence of acute knee or other lower extremity injuries. Braces did not reduce the severity of ankle, knee, or other lower extremity injuries
Babins et al. [2]	Group treated with a lace-up ankle brace and non-braced control group in high school basketball	The overall incidence of acute ankle injuries was lower in the braced group than the control group (27 vs. 78 injuries; rate, 0.47 vs. 1.41)	Acute ankle injuries among high school basketball players assigned to wear lace-up ankle braces were reduced by 68 %. The braces did not affect the severity of the injury or the rates of knee or other lower extremity injuries
Mickel et al. [33]	Prophylactic bracing or taping in high school football	There was no statistically significant difference in the incidence of ankle sprains between the 2 groups. The time required to tape an athlete averaged 67 s per ankle, resulting in a total of 97 min per ankle during an entire season, and the average cost to tape each ankle during an entire season was greater than the cost of the commercially available brace	The projected cost savings for an athletic program using prophylactic bracing could be substantial when compared with the use of prophylactic taping of the ankle

In all of these studies, braces were used to prevent ankle sprains in risk sports (basketball, football, volleyball). Three of these studies found that the use of braces reduces the incidence of ankle sprain in asymptomatic athletes in basketball and football [2, 31–33]. Mickel et al. [33] compared brace and tape use for the prevention of ankle. In this study, there was no difference in the sprain rate, but the treatment time per athlete was significantly higher in the tape group.

## Discussion

With this systematic review of the literature, we found answers to the research questions stated at the end of the hypothesis. For this purpose, we analyzed 22 articles which met the inclusion criteria.

### Surgical versus non-surgical treatment of acute ankle sprains

Today, surgical treatment plays only a minor role for the treatment of acute ankle sprains. In most narrative review articles non-operative treatment is recommended [10, 11, 14, 40].

However, a Cochrane review [26] has shown that surgical ligament reconstruction is advantageous with regard to the recurrence rate for ankle injuries, the incidence of chronic ankle problems and functional (subjective) and mechanical (objective) instability of the ankle. On the other hand, there was limited evidence for longer recovery times, higher incidences of ankle stiffness, impaired ankle mobility and more complications in the surgical treatment group. Because of the low quality of the analyzed trials, the authors concluded that there is insufficient evidence to determine the relative effectiveness of surgical and conservative treatment for acute ankle sprains.

Two later published prospective randomized trials came to similar findings. In a randomized study with a long-term follow-up, Pihlajamäki et al. [44] could show that surgery decreased the prevalence of reinjury of the lateral ligaments. A downside for surgical treatment in this study was a higher rate of II degree of osteoarthritis detected by MRI. Takao et al. [51] have shown in a randomized study with a 2 years follow-up that functional treatment alone had an approximately 10 % failure rate and a slower return to full athletic activity.

In both studies [44, 51], there was no difference in the clinical scores between surgical and non-surgical treatment.

Based on these findings, we conclude that the main advantage of surgical ankle ligament repair is that objective instability and recurrence rate was less common when compared with non-operative treatment. Balancing the advantages and disadvantages of surgical and non-surgical treatment, we conclude that the majority of grades I, II and III lateral ankle sprains can be managed without surgery. However, with regard to its advantages, surgery should not be totally abandoned. The indication for surgical repair should be made on an individual basis. We agree with van den Bekerom et al. [52] that an acute reconstruction could be indicated in athletes, because increased objective instability is a predictor for future ankle sprains [59].

Another indication for surgery could be an extensive grade III lesion of all three lateral ankle ligaments with massive hematoma [4, 42].

### Is there evidence for functional treatment or immobilization?

This question can be answered by the meta-analysis published by Kerkhoffs et al. [24]. Based on the analysis of 21 trials involving 2,184 participants these authors concluded that functional treatment appears to be the favourable strategy for treating acute ankle sprains when compared with long-term immobilization (4–6 weeks). However, these results should be interpreted with caution, as most of the differences are not significant after exclusion of the low quality trials. Many trials were poorly reported and there was variety amongst the functional treatments evaluated.

Two newer randomized controlled studies, however, found that for the treatment of grade III injuries a short period (10 days) of immobilization with a below knee cast can be advantageous [5, 6, 27].

Probably, a short period of rest in a below knee cast helps to reduce swelling and pain during the early inflammatory phase of biological ligament healing. Later during the proliferation phase and remodeling phase, immobilization in a cast could be detrimental for the healing process. According to the principle of causal histiogenesis [41] functional stress is needed for the remodeling of connective tissue. It is also well known that prolonged immobilization has a detrimental effect on muscles, ligaments and joint surfaces.

Therefore, several authors recommend that initial treatment during the inflammatory phase should be directed towards avoiding or diminishing excess swelling and ongoing injury, thus optimizing the healing process [11, 14, 53]. RICE (Rest, Ice, Compression and Elevation) therapy is considered to be the treatment of choice for the first 4–5 days to reduce pain and swelling [53] and prefer a short initial period of 5–7 days (max 10 days) of immobilization in a below the knee cast or removable boot.

### What is the most effective type of external stabilization for the treatment of acute ankle sprain?

It is generally agreed that the majority of acute grade I–III ankle sprains can be treated by non-operative measures.

During the proliferation phase, the tissue responds with vascular ingrowth, fibroblast proliferation and new collagen formation. Protection of inversion is important during this phase of healing to prevent excess formation of weaker type III collagen formation that can contribute to chronic elongation of the ligament. Controlled stress on the ligament will promote proper collagen fibre orientation. In addition, motion, stretching and strengthening will avoid the harmful effects of immobilization on the muscle, joint cartilage and bone.

We differentiate several options for external ankle protection: bandages, tape, lace up braces and semi rigid ankle orthoses. In a meta-analysis, Kerkhoffs et al. [25] have shown that the use of an elastic bandage has fewer complications than taping, but appears to be associated with a slower return to work and sport, and more reported instability than a semi-rigid ankle support. Lace-up ankle support appears to be effective in reducing swelling in the short-term compared with semi-rigid ankle support, elastic bandage and tape [25].

Newer randomized trials came to similar results. In all studies patients had better short term results with a semi-rigid ankle brace than with a bandage [5, 7, 9, 27]. Lardenoye et al. [28] compared a semi rigid brace with tape. In this study, the rate of skin complication in this group was significantly lower as compared to the tape group, but functional outcome of the ankle joint was similar between the two treatment groups, as well as reported pain. Bennyon et al. [5] showed that even for grades I and II injuries treatment with a semi rigid ankle brace combined with an elastic wrap returned subjects to normal walking and stair climbing in half the time required for those treated with the Air-Stirrup brace alone form those treated with an elastic wrap alone.

From these studies, we conclude that during the proliferation phase the ankle is most effectively protected against inversion by a semi-rigid ankle brace. For grade III injuries, the semi-rigid orthosis is adapted after the initial short immobilization phase.

All studies about non-surgical treatment of ankle sprains have one methodological flaw, because they report only short-term follow-up data and no resprain rates. Malliaropoulos et al. [29] reported with a cohort study a resprain rate of 17.8 % at 2 years after non-operative ankle sprain treatment. Because of this methodological flaw, we do not know the resprain rates of different types of different types of external support for the treatment of ankle sprains. More prospective randomized studies with a longer follow-up are needed to answer this question.

### Is there any evidence for neuromuscular training for treatment of acute ankle sprains?

In 1965, Freeman [15, 16] hypothesized that balance and coordination training could diminish proprioceptive deficits associated with ligamentous injury to the ankle. Contemporary theory suggests that balance and coordination training may have both local and central effects on the sensorimotor system [19, 45, 46]. However, consensus is lacking regarding the clinical evidence of the efficacy and effectiveness of these interventions. In contrast to the hypothesis of Freeman, a previously published systematic review [40] reported that there was no evidence for effectiveness of physiotherapy as a treatment strategy for acute ankle sprains.

Two randomized controlled trials published later, however, reported fewer resprains after 12 months follow-up [20, 62].

Even the results of the studies analyzed in this systematic review are contradictory. Van Rijn et al. [56] found that conventional treatment of an ankle sprain combined with supervised exercises as compared to conventional treatment alone after an acute lateral ankle sprain does not lead to differences in the occurrence of resprains or in subjective recovery. Bleakley et al. [6] could also detect no difference in the resprain rate between the groups with and without exercise after an acute ankle sprain. However, this study showed a positive effect of exercise for improved ankle function and activity. The 2Fit study, however, showed a positive effect of a non-supervised home-based proprioceptive balance board training program in addition to usual care on the resprain rate Hupperets [22]. A process evaluation showed that only 23 % of the intervention group indicated to have fully adhered with the neuromuscular training program. Significantly fewer recurrent ankle sprains were found in the fully adherent group compared with the group that was not adherent [61]. This could be an explanation for the missing effect in the studies conducted by van Rijn [56] and Bleakery et al. [6]. The power of these studies was with 102 participants each much lower than in the 2 Fit trial with 522 participants.

In conclusion, based on the high level 2 Fit study, we conclude that balance training can be used after an acute ankle sprain in an effort to reduce future ankle sprains.

### Is there any evidence for neuromuscular training for prevention of acute ankle sprains?

A meta-analysis about the prevention of ankle sprains published in 2001 [18] found that there was limited evidence for reduction in ankle sprain for those athletes with previous ankle sprains who did ankle disk training exercises.

In this systematic review, we analyzed only studies published between 2002 and 2012. Two of the randomized trials that met the inclusion criteria focused on the primary preventive effect of balance training. In these studies, the ankle sprain rate was significantly lower after balance training only in the group of athletes with a previous sprain [30, 58]. In players without a history of ankle sprains, there was just a tendency towards a lower injury rate in the training group. These studies confirm results of previous studies which were published before 2002 [48, 50]. A sensitivity analysis of Verhagens prevention study [60] showed that only a balance board training program aimed at players with the previous ankle sprains could be cost-effective over a longer period of time.

Probably, more well-designed prospective studies with larger samples are needed to show a significant effect also for athletes without a previous ankle sprain. However, even if these studies could show an effect, the number to treat is expected to be high.

These findings can be explained since the most important risk factor for an ankle sprain is a previous ankle sprain [13]. This might be due to reduced proprioceptive function [35, 36, 38]. Mitchell et al. [35, 36] have demonstrate a slower reaction time and postural sway deficits in ankles with functional instability. These authors concluded that individuals, who sustain an acute ankle sprain and those with functional instability require rehabilitation that improves proprioception, strengthens the evertors and dorsiflexors, and restores peroneal reaction time.

In conclusion, in accordance with a previous systematic review [39], the articles published between 2002 and 2012 provide evidence that a balance training can be used in an effort to reduce future ankle sprains in athletes with a previous injury.

### Is there any evidence of brace use for the prevention of ankle sprains?

Three of the four identified studies showed that the use of lace up braces reduced the incidence, but not the severity of acute ankle injuries in football and basketball players [2, 30, 31]. One study compared prophylactic bracing or taping in high school football [33]. In this study, there was no difference in the rate of ankle sprains between the two groups. However, the cost analysis showed that tape use was less cost-effective (more time) than brace use.

These results confirm results of a meta-analysis which was published in 2001 [18]. This meta-analysis provided good evidence for the beneficial effect of ankle braces to prevent ankle sprains during high-risk sporting activities (e.g. soccer, basketball).

In conclusion, there is good evidence from high level randomized trials in the literature that the use of a brace is effective for the prevention of ankle sprains.

### Limitations of this systematic review

Every effort was made to obtain level one evidence studies to answer our research questions. However, even these high quality studies vary in terms of quality of methodology and reported outcomes.

The problem of the meta-analysis was that many studies that were included had methodological flaws. Therefore, in none of the meta-analysis included in this review, the authors found strong evidence for one of the examined treatment options.

The RCTs about surgical versus non-surgical treatment have an adequate follow-up (2–14 years), but a low number of patients. Therefore, the power of these studies might not be large enough to find out any differences in clinical scores. On the other hand, the RCT about non-surgical treatment of acute ankle sprains have large case numbers, but the follow-up varies between 9 and 12 months. This period is too short to examine resprain rates. Therefore, none of the RCT about non-surgical treatment with external support reports the rate of recurrent sprains. The papers originate from different countries and therefore, may not be applicable in every aspect to all populations. Limiting the review to English language articles only has the risk for high-quality non-English articles to be excluded. We looked only at studies which recruited adults. Therefore, the results are not applicable for the treatment of ankle sprains of children (i.e. 18 years or older).

Owing to the limitation of our search RCTs about pharmacolocgical treatment of ankle sprains were excluded. This was done to limit the scope of this systematic review. Many studies deal with the use of NSAIDs, for example. A new treatment option is the use of hyaluronic acid injections that should be associated with a more rapid return to sport and with only a few associated adverse events, but the relative increased cost of this treatment versus the standard of care has to be considered [43].

### Future directions

Despite the existing evidence from meta-analysis and RCT, many patients develop chronic problems after injury of the ankle ligaments [17, 29, 55, 57]. Therefore, there is reason to believe that many question for the treatment of ankle injuries are still unsolved. These unresolved issues include time and criteria for return to sports, duration of ankle protection by external support, use of and diagnostics of associated injuries.

## Conclusions

Balancing the advantages and disadvantages of surgical and non-surgical treatment we conclude that the majority of grades I, II and III lateral ankle ligament ruptures can be managed without surgery. The indication for surgical repair should be always made on an individual basis. This systematic review supports a phase adapted non-surgical treatment of acute ankle sprains with a short-term immobilization for grade III injuries followed by a semi-rigid brace. Types I and II injuries might best be treated with a semi-rigid brace. Neuromuscular training should support functional rehabilitation after ankle sprain. Balance training is effective for the prevention of resprains of athletes with previous sprains. Braces are also effective for the prevention of ankle sprains in athletes. More prospective randomized studies with a longer follow-up are needed to find out what type of non-surgical treatment has the lowest resprain rate.
